# “A peer support worker can really be there supporting the youth throughout the whole process”: a qualitative study exploring the role of peer support in providing substance use services to youth

**DOI:** 10.1186/s12954-023-00853-3

**Published:** 2023-08-29

**Authors:** Roxanne Turuba, Ciara Toddington, Miranda Tymoschuk, Anurada Amarasekera, Amanda Madeleine Howard, Violet Brockmann, Corinne Tallon, Sarah Irving, Steve Mathias, J. L. Henderson, Skye Barbic

**Affiliations:** 1 Foundry, 915-1045 Howe Street, Vancouver, BC V6Z 2A9 Canada; 2 Providence Health Care, 1081 Burrard Street, Vancouver, BC V6Z 1Y6 Canada; 3 Providence Research, 1190 Hornby Street, 10th Floor, Vancouver, BC V6Z 2K5 Canada; 4 Faculty of Medicine, University of British Columbia, 317-2194 Health Sciences Mall, Vancouver, BC V6T 1Z3 Canada; 5 Department of Occupational Science and Occupational Therapy, University of British Columbia, 317-2194 Health Sciences Mall, Vancouver, BC V6T 1Z3 Canada; 6 Centre for Health Evaluation Outcome Sciences, 588-1081 Burrard Street, Vancouver, BC V6Z 1Y6 Canada; 7 Centre for Addiction and Mental Health, 80 Workman Way, Toronto, ON M6J 1H4 Canada; 8 Department of Psychiatry, University of Toronto, 250 College Street, 8Th Floor, Toronto, ON M5T 1R8 Canada; 9 Youth Wellness Hubs Ontario, 80 Workman Way, Toronto, ON M4J 1H4 Canada

**Keywords:** Peer support, Youth, Adolescents, Young adults, Substance use, Qualitative research, Participatory action research

## Abstract

**Background:**

Youth (ages 12–24) rarely access services and supports to address substance use concerns. Peer support can facilitate service engagement and has been associated with positive substance use recovery outcomes in adults, yet few studies have examined this role among youth specifically. As such, this qualitative study explored the role of peer support in providing substance use services to youth in British Columbia and how best to support them in their role.

**Methods:**

Participatory action research methods were used by partnering with youth who had lived/living experience of substance use, including peer support workers, to co-design the research protocol and materials. An initial focus group and subsequent interviews were held with 18 peer support workers who provide services to youth (ages 12–24) based on their own lived experience with mental health and/or substance use. The discussions were audio-recorded, transcribed verbatim, and analysed thematically using an inductive approach.

**Results:**

Peer support workers' core experiences providing substance use services to youth centred around *supporting youth throughout the whole process*. This was accomplished by meeting youth where they are at, providing individualized care, and bridging the gap between other services and supports. However, participants experienced multiple organizational barriers hindering their ability to support youth and stressed the importance of *having an employer who understands the work you are doing*. This involved having someone advocating for the peer support role to promote collaboration, empowering peers to set boundaries and define their own role, and providing adequate training and mentorship. Finally, peer support workers described how their *lived experience bridges connection and de-stigmatization* at the individual, organizational, and community level, which was unique to their role.

**Conclusions:**

Peer support plays a unique role in youths’ substance use journeys, given their own lived experience and flexibility within their role. However, their position is often misunderstood by employers and other service providers, leaving peers with inadequate support, training, and mentorship to do their job. The findings from this study call for improved integration of peer support into service environments, as well as standardized training that is in-depth and continuous.

## Background

Addressing substance use concerns among adolescents and young adults is an important priority given the evidence linking early onset to substance use and mental health disorders later in life [1–3]. Youth (ages 18–25) report the highest prevalence of substance use (including alcohol, cannabis, and illicit substances) in North America; however, they are unlikely to access substance services as they often do not perceive a need for support and/or treatment [4]. Evidence suggests that youth also experience barriers accessing substance use services due to privacy and confidentiality concerns [5], fear of stigmatization [6–8], and treatment goals that do not match the way current services are delivered [7, 9, 10].

Peer support provides individuals with lived experience an opportunity to shape how substance use services are delivered and has been found to be effective in reducing substance use stigma among clinical colleagues. Working closely with people with lived experience can help shift non-peer colleagues’ pre-conceived notions about people who use substances and thus improve relationships between clients and service providers [11, 12]. The peer support role has been implemented in various substance use care settings, including in community (e.g. harm reduction services, housing support] [13], inpatient [14, 15], emergency departments [16], the criminal justice system [17], and specialized treatment and recovery settings [18]. It has been associated with positive recovery outcomes in adults, such as increased self-confidence and self-esteem; reduced substance use, relapses, and hospitalizations; smoother interactions with family members; and improved overall treatment experiences [12, 19, 20]. Further, the role has been found to facilitate engagement with “hard-to-reach” clients that may be hesitant about accessing treatment and support for mental health and substance use concerns [21, 22] and could therefore be an effective way to engage youth [23]. Barton and Henderson [23] argue that peer support may also help foster supportive relationships with youth, given their own lived experience, and promote recovery through observed behaviour.

Although peer support workers are generally defined as people who support individuals who share similar lived experiences by providing emotional, informational (e.g. life skills), instrumental (e.g. employment support), and/or affiliational (e.g. social connection) support [19, 24], their roles and responsibilities are often not well defined and can vary widely across organizations and service settings [12, 20, 23]. While some services integrate peer support within clinical care teams, other organizations are entirely or partially led and delivered by peers, where their role is independent of other services and providers [25]. For example, peers working within a tertiary care setting may attend hospital appointments with the physician or nurses and act as a mediator [15], while peers providing harm reduction services may provide support in a community setting, outside of interactions with other service providers [26].

While there is growing evidence exploring the role and benefits of peer support to address substance use concerns in adult populations [18, 20, 27], few studies have explored its role to support youth, who may especially benefit from this intervention given the reduced power differential between youth and peers as compared to traditional services delivered by health professionals. As such, this qualitative study aims to understand the role of peer support in providing substance use services to youth and how best to support them in this role.

## Methods

### Study design and setting

This study is part of a multi-phase project entitled *Building capacity for early intervention: Increasing access to youth-centered, evidence-based substance use and addictions services in British Columbia and Ontario*, which aims to create youth-informed substance use training for peer support workers and other service providers working within an integrated services model. The project is being led by two integrated youth service (IYS) hubs in British Columbia (BC) and Ontario. BC has a population of approximately 4.6 million people who predominantly identifies as European white settler, followed by East Asian, South Asian, Indigenous, and South Asian peoples [28]. Nationally, BC has been disproportionately impacted by the drug toxicity crisis, counting 2,272 illicit toxicity deaths in 2022 alone, largely due to the increasingly toxic drug supply [29]. Although more than half of BC’s population reside in the Metro Vancouver area, similar rates of illicit toxicity deaths are found across health regions [29].To support the development of substance use training in BC, the BC project team conducted two qualitative research studies (Phase 1), titled *The Experience Project*, to better understand how youth perceive and experience substance use services in BC (published elsewhere [8]) and understand the role that peer support workers play in youths’ substance use journeys and how best to support them. This paper focuses on the Phase 1 study exploring the role of peer support in providing substance use services to youth and follows the Standards for Reporting Qualitative Research (SRQR), a 21-item checklist for reporting qualitative research [30].

Participatory action research (PAR) methods [31, 32] were used to guide the multi-phase project by using varying levels of PAR principles across each phase. We worked collaboratively with youth who had lived/living experience of substance use, including peer support workers, to explore the experiences of youth and peer support workers accessing/providing youth substance use services (Phase 1) and improve these experiences by developing youth-informed substance use training based on the Phase 1 study findings and their own lived experiences (Phase 2). In Phase 1, we created a project youth advisory committee title the Youth4Youth (Y4Y), which comprised of 14 youth (under the age of 30) who had lived and/or living experience of substance use and resided in BC at the time of the study. The Y4Y was responsible for co-creating and revising the research protocol and materials and asked to ensure their relevance to youth and peer support workers, while identifying gaps. Bi-weekly meetings were held over Zoom between the research team and the Y4Y to discuss their input and reflect on various matters, such as important research questions, safety measures, and cultural and identify considerations to enrich the research process. These discussions promoted self-reflection and learning among the research team and helped us identify ways to reduce power dynamics between researchers, youth partners, and study participants, and work towards decolonizing our research practices. For instance, to minimize power dynamics between adult researchers and youth, three Y4Y members were hired as youth research assistants to undertake data collection, validate the thematic analysis, and support with knowledge translation. Feedback from youth also led to organizational policy changes to facilitate meaningful youth engagement and minimize harmful practices. Additional details about the Phase 1 methods have been published elsewhere [33].

### Participants

To be eligible, participants had to provide peer support services to youth (defined as ages 12–24) based on their own lived experience with mental health and/or substance use and live in BC at the time of consent. We relied on social media and targeted advertisements as the main methods of recruitment. Organizations that provide peer support services across the province were contacted about the study and helped share recruitment adverts. Interested peer support workers contacted the research coordinator (author RT) to confirm their eligibility. A phone call was scheduled with each participant to go over the consent form and obtain verbal consent. The research coordinator filled out the consent form on their behalf and sent them a signed copy for their records.

### Data collection

Data collection began in November 2020 and continued until May 2021. Before participating in a focus group or interview, participants were asked to voluntarily complete an anonymous demographic survey. Focus group/interviews questions revolved around peer support workers’ experience supporting youth with substance use concerns, their recommended approach, how prepared they felt, and what their role was compared to other colleagues. We also asked participants what would constitute an optimal working environment for them and how to address barriers youth face when accessing substance use services (e.g. family dynamics, identity, culture, stigma). After conducting an initial semi-structured 2-h focus group with three peer support workers, we changed our data collection methods to individual in-depth interviews (*n* = 15), which lasted 30 min to an hour. This adjustment was in response to the level of variability within the peer support role, as well as recruitment challenges, which made it difficult to schedule focus groups in a timely manner. Each session was facilitated by two trained research team members, including a youth research assistant with lived/living experience. Discussions were conducted virtually over Zoom. Participants were provided with a $30 or $50 honorarium for taking part in an interview or focus group, respectively.

### Data analysis

The focus group and interviews were audio-recorded and transcribed verbatim by a professional transcriber and then revised by the research coordinator. Transcripts were then coded using NVivo (version 12) and thematically analysed by the research coordinator following an inductive approach using Braun and Clarke’s six-step method [34, 35]. This involved reading the transcripts multiple times while taking initial memos and reflections. A data-driven approach was used to generate verbatim codes and identify semantic themes. Meetings were held with the youth research assistants who facilitated the interviews (authors AA, AMH, and VB), and two peer support workers from the Y4Y advisory (first author CT and MT) to discuss the relationships between the codes and potential sub-themes and overarching themes. This involved reviewing and refining the themes and selecting supporting quotes to highlight in the manuscript in order to strengthen the credibility and validity of the findings.

## Results

We interviewed 18 peer support workers in total. Sociodemographics and peer support experience are listed in Table 1. Participants’ median age was 23 and primarily identified as women (92.9%) and white (57.1%). Half the participants worked at an IYS centre, while others worked in a variety of care settings (e.g. community and school settings, crisis support, mental health service). Years of experience as a peer support worker varied, ranging from one month to 10 years (median = 2.5 years).

**Table 1:** Characteristics of peer support worker participants

Characteristics	Participants *N* = 14^a^
*Socio-demographics*	*N* (%)/Median
**Age**	
Age (Median)	23
**Gender**	
Woman	13 (92.9)
Genderfluid	1 (7.1)
**Ethnicity**^b^	
White	8 (57.1)
Chinese	2 (14.3)
South Asian	2 (14.3)
Southeast Asian	1 (7.1)
Fijian	1 (7.1)
**Employment**^c^	
Integrated youth service (IYS)	9 (50.0)
Community setting	3 (16.7)
School setting	3 (16.7)
Crisis support	2 (11.1)
Mental health service	1 (5.6)
**Received peer support training**^c^	
Yes	15 (83.3)
No	3 (16.7)
**Experience as a peer support worker**^c^	
Under 1 year	3 (16.7)
1–2 years	5 (27.8)
2–4 years	6 (3.3)
5–10 years	4 (22.2)

^a^The demographic survey was voluntary. Response rate was 78% (14/18 completed)

^b^Participants could select more than 1 response. Therefore, the number of responses may be greater than the total number of participants who completed the survey

^c^All 18 participants responded to this question during the interview

**Fig. 1:**
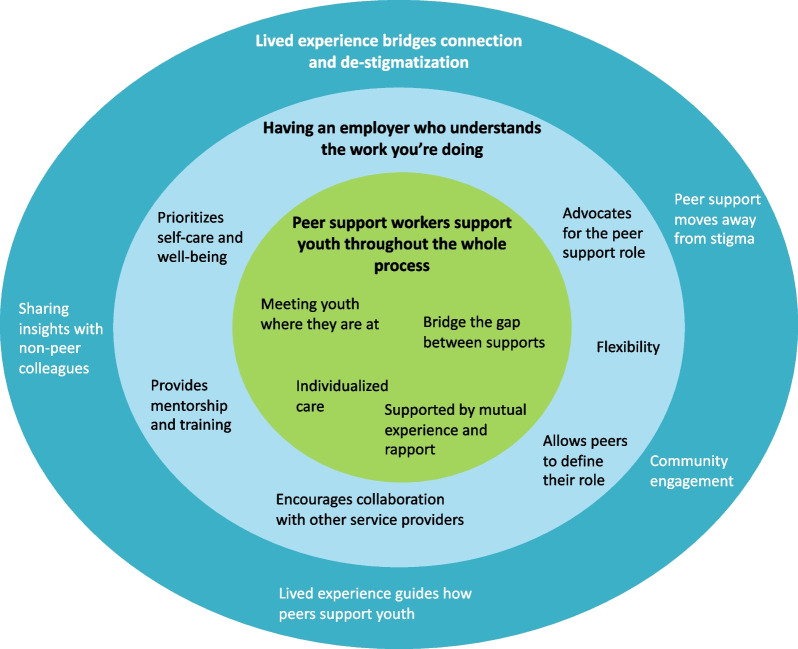
Overarching themes describing peer support workers' experiences supporting youth who use substances

The qualitative analysis led to the identification of three overarching themes that describe peer support workers’ experiences supporting youth with substance use concerns (see Fig. 1). This centred around the first theme wherein peer support workers support youth throughout the whole process, by meeting youth where they are at, providing youth with individualized care, and bridging the gap between other services and supports, which was supported by their own lived experience and ability to build and maintain strong relationships with youth. However, the second theme describes how numerous organizational barriers hindered their ability to succeed in their role and the importance of having an employer who understood the work that they were doing. This included empowering peers to set boundaries and define their own role, providing them with adequate training and mentorship, and advocating for the peer support role to help other service providers understand their responsibilities and work collaboratively to support youth. Finally, the last theme discusses the wider impact of hiring peer support workers and how their lived experience plays an integral role in their ability to connect with youth and de-stigmatize substance use at an individual, organizational, and community level. The next sections will focus on describing these three overarching themes and supporting quotes in further detail.

### Peer support workers support youth throughout the whole process

Peer support workers described their role being centred around meeting youth where they are at, which occurred throughout the entire care continuum, contrary to other service roles: *“A peer support worker can really be there supporting the youth throughout the whole process, throughout the visit with the doctor or throughout the counsellor and I guess, guiding them through their treatment…or guiding them through their experience”* (P12). As such, peer support workers helped youth with whatever they might need in that moment, with the goal of keeping youth safe rather than solely focusing on abstinence. This involved active listening, emotional support, providing harm reduction supplies and education, and helping youth identify and connect to services.

This involved using an individualized approach when supporting youth by considering the factors impacting youth’s substance use (e.g. trauma, mental and physical illness, life transitions, etc.), their environment, who their supports are, and their service goals. For instance, this meant *“respecting that not everybody wants to stop [using substances] or can stop”* (P02) and acknowledging that removing substance use as a coping mechanism could cause more harm. Further, although having support from family and friends was often described as a *“game-changer”* (P16) for youth seeking recovery, peer support workers acknowledged that this was not always possible or helpful depending on their relationship with family and friends and their cultural norms towards mental health and substance use.

Thus, participants highlighted the importance of developing a strong relationship with youth that was built on respect and trust to truly understand where youth were coming from and what they needed to feel safe and supported. This meant coming from a place of empathy and compassion, *“creating a safe environment for them where they’re not feeling judged”* (P06) and letting them *“take the lead”* (P15), such as directing the type of support they wanted to receive, deciding if/when to involve family and friends, and *“give them a sense of empowerment that is taken away from so many of them”* (P11). Participants reported that connecting with youth also involved practicing cultural humility and safety in the workplace, by acknowledging generational trauma, cultural norms, and gender identities. This was described as crucial in maintaining trust with their clients: *“I would say too like for trans youth, if there’s not competency and literacy around using the right name and right pronoun like that’s where that care and relationship ends. Like as soon as they’re not being addressed respectfully and properly the trust is ruptured so quickly”* (P02).

Peer support workers’ ability to connect with youth was closely related to their own lived experience, which allowed them to understand what youth were going through and provide authentic, compassionate, and non-judgmental care. This in turn allowed youth to feel more comfortable discussing difficult topics without fear of repercussions or judgment, and enabled peer support workers to understand what was really happening for the youth they were supporting, including the reasons for their substance use and how best to support them from a holistic perspective, such as addressing their basic (e.g. safety, stable housing, food security) and complex (e.g. mental health condition, trauma) needs, building their natural support system, and connecting them to other services and supports:

*“I think for me, like what [participant 2] said in terms of making the gap smaller, like focusing on relationships I guess, like kind of bringing a more human element to accessing care...it’s pretty common for a doctor or a counsellor to say, “Wow that youth is usually really, really difficult to communicate with, how did you connect with them” or “How did you get that information?” It’s like, we just talk to them like they were people.”* (P01)

This quote demonstrates the unique insights peer support workers have on youths’ experiences and needs and the opportunity for other service providers to learn from peers on how best to support youth and improve their care experiences. Peer support workers’ role was described as unique from other health care providers as it gave youth a safe place, slightly outside the clinical care team, to discuss what they wanted in that moment, rather than having a concrete agenda. As one participant describes: *“People on the care team are there to help the youth but they’re still monitoring them in the clinical context. So yeah, I think it’s nice to have – for that youth to have a breath outside where it’s still a supportive adult they can trust”* (P06).

Peer support workers described how connecting youth to appropriate services and supports to address their substance use concerns (e.g. counselling, primary care, harm reduction) was also an integral part of their role, which was facilitated by their own experiences of accessing services and understanding what supports and services were helpful to them and other youth they supported. This involved helping youth find and navigate the right services, advocating for them, discussing how their other appointments were going, providing harm reduction supplies, teaching youth how to administer naloxone, and helping them get to appointments. It also involved identifying whether and when to connect youth to their natural support systems, including friends, family members, elders, and community, and helping them navigate those relationships: *"I would say handing out harm reduction supplies, having those heart-to-hearts, connecting them with their elders, recognizing when they need to go to the hospital and they need medical attention that I can’t provide.”* (P11).

### Having an employer who understands the work you are doing

Having *“an employer who understands the work that you are doing”* (P11) and valued peer support workers’ lived experience was crucial for peer support workers to meet youth where they are at and feel confident in their role. This included recognizing the *“emotional toll”* (P11) peer support workers encountered using their lived experience to support youth with substance use concerns and prioritizing peer support workers’ well-being. As such, participants stressed the importance of setting boundaries in terms of the type of support they were willing and able to provide, which involved defining their own role with their employer based on their lived experience, expertise, and skills, and developing safety plans for when difficult situations arose:

*“Because of our experience, that brings so much to the table, but it also means that you have someone on your team, like for a lack of a better term, that isn’t like everybody else. So I think it’s really important to recognize that and kind of work with them to understand what their boundaries are and what they want their role to look like, and what they bring to the table...Like in my experience, it’s been clear – good in the way that, I know that if I’m uncomfortable in a situation, I can have a counsellor or something come into a conversation and help me work through it. But I don’t always think that they really understand what it’s like from our perspective in terms of our own experience and how that can affect how we support youth.”* (P15)

Having opportunities to regularly debrief and check-in with supervisors and/or colleagues was described as one of the biggest forms of support, as peer support workers often *“learned on the job”* (P06). This involved getting other members of the care team’s perspectives on how best to support clients, sharing resources, and learning from each other, which was often done informally or during weekly team meetings and group text chats. This not only created a sense of community within the workplace but also encouraged *“self-care and work-life balance”* (P08) so that peer support workers could operate *“from a place of being present and being able to extend yourself for others, because this work can be very draining and take a lot from you”* (P13).

Having a manager or supervisor who understood that mental health and substance use disorders are chronic conditions that people continue to deal with also allowed participants to discuss and work through personal challenges rather than allowing them to impede their ability to do their job. As one participant describes after having a relapse:

*“...it did come to a place where I felt it was necessary to tell my managers at the time what had happened and how we should move going forward. I’m just personally so incredibly grateful that [my employer] chose to support me and let me continue my work as a peer support worker. Because if I would have lost [my job] because of what I lived with, that would’ve just created all these new feelings of shame, and guilt, and my community allowed me to come back, start from where I was at, and just continue and just try again.”* (P16)

This quote demonstrates the importance peer support workers placed on having an employer who understood their lived experience and worked with them to determine the type of support they were comfortable and willing to provide. This flexibility within their role was also described as facilitating their ability to build strong relationships with youth and truly meet them where they are at. This included having the ability to take their time to connect with youth without having to adhere to a specific agenda and incorporate humour and fun activities such as icebreakers, walks, car rides, and spending time with an animal:

*“One of my biggest assets with my job is I have this creative flexibility to work with kids on where they’re at. And coming from that place of just empathetic, non-judgmental understanding where I can relate to the kids on the core emotions…If youth are feeling safe with me and that they can talk about these things for maybe the first time ever, I think that’s kind of the optimal environment for promoting change and giving people a chance to really go for recovery.”* (P13)

Although their lived experience guided how they assisted youth, peer support workers stressed the importance of mentorship and proper training. This involved having access to resources and supports at work (e.g. counselling services, wellness programs), and being provided with a manageable workload and proper compensation. Yet, many peer support workers described not feeling adequately prepared to support youth with substance use concerns and expressed the need for more training on boundary setting, self-care, healthy coping mechanisms, and substance use-specific training in order to handle a wide array of situations that could occur. This included training on how to deal with risky situations, crisis responding, harm reduction, and cultural safety trainings to enable them to better support youth and families from different cultural backgrounds.

Although most participants had received some sort of training (83%), these varied widely across organizations and additional trainings on specific health issues (i.e., trauma, mood disorders, substance use and concurrent disorders, suicide prevention, violence prevention, eating disorders) were often not provided and had to be sought out independently. Further, some participants described not being provided with essential information to perform their day-to-day activities, such as basic information about work processes (i.e., reporting structures, administrative tasks, and ethical workplace practices) and the model of care used within their employment setting, creating significant barriers for peers to perform efficiently.

The type of training provided also influenced how supported participants felt. Interactive training methods were described as most helpful “*to get some practice in terms of what [providing peer support] could really look like and what skills to bring*” (P15). This included acting out scenarios, learning from other peer support workers and guest speakers, engaging in discussions with peers, in-depth specialized training lessons (e.g. 2SLGBTQIA+, substance use, harm reduction, suicide ideation), and hands-on experience facilitated through shadowing. As best practices continue to change, peer support workers highlighted the need for continuous training to remain up to date.

Having an employer who advocated for the peer support role was key to incorporating peer support into clinical practice and ensured that other service providers within the care team understood what their position entailed and minimize the power dynamic over peers. Having colleagues who understood peer support roles promoted collaboration with peer support workers, who were seen and treated as valued members of the care team: *"It was less like can you do this for me and more like here’s what’s going on with this person, what do you think?"* (P01). However, participants described how their role was not always appreciated or valued by non-peer colleagues, particularly when peer support was not well understood by other members of the care team. This put peer support workers in difficult situations when being asked to do things outside of their job description due to existing power dynamics between non-peer colleagues and peer support workers:

*“I think that we’re still at an interesting point in history for peer support work where people don’t know what it is and what it’s for so it’s very unprecedented. In the organization, if there hasn’t been somebody doing peer support there already, I think there really needs to be someone on the inside really fighting the good fight and really pushing for it and advocating for it. Because yeah, it’s hard to get it up off the ground otherwise, I think. I think also it could just be super beneficial to have that champion for peer support workers just to remind the clinicians and other staff that this is the peer support workers role, this is what they’re gonna do, this is outside of their role, so if they have free time and they want to do it they can but by no means is it their job to do that. Because I know sometimes clinicians can, you know, just think of peer support worker as MOAs [medical office assistants] or people to run around and do things for them but that’s not true at all. We are our own people with our own things to do.”* (P02).

### Lived experience bridges connection and de-stigmatization

Peer support workers could relate and empathize with youth given their own lived experience, which gave them insight on the various barriers that hindered their ability to access appropriate substance use support (e.g. lack of specialized services and culturally safe and inclusive service options, long waitlists, transportation barriers, challenges addressing basic needs, ageing out). Judgment from community, family members, and service providers was described as one of the biggest barriers hindering youths’ ability to access and receive support, which was compounded by discrimination (e.g. racial, ethnic, gender). This perspective guided the way peer support workers interacted with youth, which was described as coming *“from a place of love and compassion”* (P08) and *“understanding that they are struggling and just knowing they’re in a bad place and it’s not their fault”* (P18).

Having someone provide care based on their lived experience played a large role in de-stigmatizing substance use for youth accessing services. Knowing someone who had gone through it helped youth *“know it’s not just them”* (P08) and showed them that recovery was possible. This also created a safe space for youth to open up and share their struggles without fear of judgment. The role also de-stigmatized peer support workers own experiences, as it gave their lived experience value and meaning and the ability to give back to those in similar situations. As one participant describes:

*“For me, peer support was really a gamechanger in the sense that I didn’t have to hide from who I was. When it became an asset, when my journey was something that was appreciated and was useful to other people, that was a big turning point in my journey. Because no longer was it about this shame and “guiltful” past that I had to withdraw from in order to move forward. I think there’s a lot of discussion on how we use our journeys and the story that we tell ourselves, the narrative behind our past and our lived experience. When I gave myself permission to be open and vulnerable, and start using that in my peer support practice, I noticed that it gave other people permission to do the same. And that really is what opens up this very direct and honest communication between me and the peers I see.”* (P16)

Peer support workers also played a role in de-stigmatizing substance use among their non-peer colleagues by sharing their insights on how service providers could improve how they approached youth. Suggestions included acknowledging the power they held over youth, practicing humility, and working collaboratively with youth to meet their individual needs. As one participant describes:

*“To recognize their place, the power dynamic of the youth coming in versus them, in between that, and the privileges that they hold that the youth coming in might not. Because you can have empathy and you can be a kind person. But sometimes, service providers forget to listen, to really try to listen and understand”* (P18)

Participants described various ways services and service providers could improve youths’ access to services, such as collaborating with youth and peers with lived experience when creating services, providing wrap around care, having integrated services located in one building, supporting youth regardless of age, and advocating for more culturally safe spaces, such as incorporating more multi-cultural workers and practices. This also meant ensuring organizational policies took a harm reduction approach rather than causing more harm. As on participant describes barriers with treatment facility policies:

*“You typically don’t have access to phones for the first 4-6 weeks when you go into a centre. That kid has lost another contact and another support in their life. I don't think that's good, and I didn't feel prepared for that because when a kid does call you after that 6 weeks, they're like, "Where were you? How come you didn't call? How come you didn't do anything to support me?”* (P11).

Finally, peer support workers played a role in reducing social stigma surrounding substance use through community engagement. This involved sharing their stories and the services they provide through social media, in schools, and with the broader community. *“Giving voices to those that have or are struggling through it, to have them speak out and share their stories is really powerful in decreasing barriers”* (P10). They also shared ideas to reduce social stigma, such as providing more appropriate (e.g. non-stigmatizing) substance use education in schools for youth and medical professionals in training and investing in de-stigmatization campaigns.

## Discussion

Peer support workers described playing a unique role in youths’ substance use journeys, given their own lived experience and flexibility within their role. Participants emphasized how this fostered connection and trust and enabled them to truly meet youth where they are at throughout the entire care continuum. Peers also reflected on the multifarious barriers that hindered youths’ ability to access substance use services and described how their role could reduce some of these obstacles. However, they noted how their role was often misunderstood by employers and other service providers, which left them with inadequate support, training, and mentorship and hindered their ability to support, not only youth, but themselves.

The findings from our study suggest a need for better integration of peer support across health care settings to empower peers to succeed in their role, specifically when it comes to developing and implementing substance use services for youth. To do so, participants proposed having peer support champions at leadership levels, not only to educate other staff members about their role and promote collaboration, but to ensure peers have someone supporting them who truly understands the work they are doing. A systematic review exploring peer support worker experiences in adult substance use treatment settings echoed the lack of integration of peer support programs into traditional treatment models, which led to experiences of exclusion, tokenism, and stigma from other colleagues [18]. Unclear job descriptions also influenced peer support workers’ ability to shape their role based on their own boundaries and expertise, which impacted their self-confidence and overall recovery. This further highlights the need for a system-level approach providing multidisciplinary training about the peer support role and their scope of work, in order to fully integrate peer support into previously conservative models of care.

Additionally, participants described that without adequate support and training, they were at greater risk of distress and burnout. Peer support workers expressed a need for continuous comprehensive training, ongoing mentorship, and opportunities to learn from peer mentors, which is consistent with other studies [11, 12, 15, 18]. Inconsistent training experiences among participants also suggest a need for standardized training on the basics of peer support, self-disclosure, and boundary setting. This should be complemented with information about organizational workflows and processes to ensure peers can conduct day-to-day tasks and understand how to work with other staff members. Additionally, participants felt they would benefit from in-depth training sessions to support youth with complex needs, including substance use, trauma, mental health, and cultural safety, given their broad scope of practice.

People who use drugs have long advocated for a harm reduction approach to address substance use concerns, including the involvement of people with lived/living experience in the design and delivery of services [36]. As shown from this study, peer support workers have unique insights on the complexities associated with substance use and recognize the need for an individualized and trauma-informed approach, while building and maintaining strong relationships with youth and allowing them to direct their own care. These findings resonate with studies exploring youth experiences with substance use services, including harm reduction services, opioid agonist treatments, and overall substance use services [6–8, 37]. A study of youth in BC also affirmed feeling more at ease talking to someone who had similar lived experiences and appreciated a more holistic approach to care which considered all aspects of their lives [8]. This suggests that peer support workers and youth have similar priorities when addressing substance use concerns, which is not surprising given their mutual lived experience. Peer support therefore presents a unique learning opportunity for organizations and service providers to better understand the needs and experiences of youth who use substances and increase youths’ autonomy to make decisions in their own care. Peer support workers from this study described ways service providers could approach youth and improve their relationships with them, which further strengthens this point. They also described playing a role in educating the wider public about substance use and de-stigmatizing youths’ experiences, highlighting multiple advantages to involving peers with lived experience in service design and delivery, and bridging practice to policy. Although there is growing interest with involving peers in policy and program development, peers remain largely underutilized [38, 39].

While the findings from this study have important implications in the way youth peer support services are implemented and integrated to support youth with substance use challenges, there are limitations to consider. Our findings represent the experiences of peer support workers who mainly identify as white women. Although women make up most of the health and social service workforce [40], the findings may have varied with a more diverse participant sample. Further, this study explores the role of peer support within a publicly funded health care system in Canada, which may not entirely translate to other healthcare systems. Given the lack of literature exploring the role of peer support in providing substance use services for youth, this study explored the experiences of peers who worked in a variety of service settings and had varying levels of exposure supporting youth who use substances. As such, future studies exploring these differences are important as different considerations and integration approaches may be warranted. Additionally, gaining the perceptions of supervisors and other clinical service staff who work with peer support workers could provide important context regarding the barriers to integration. Exploring community differences may also help better understand how the peer support role may differ across rural and urban communities.

## Conclusion

This study highlights the important role peer support workers play in youths’ substance use journeys and delivering patient-centred care, given their own lived experience and flexibility to meet youth where they are at. A lack of integration and standardized peer support training are barriers to ensuring access to these evidence-based youth services. These findings emphasize a need for a system-level approach to fund and integrate peer support services into existing models of care and establish meaningful mechanisms to ensure involvement of peer support workers in youth substance use service design, delivery, education, and policy.

## Data Availability

The datasets generated and analysed during the current study are not publicly available due to the potential for identifying participants but are available from the corresponding author on reasonable requests.

## Abbreviations

IYS: Integrated youth services
BC: British Columbia
PAR: Participatory action research
Y4Y: Youth4Youth
2SLGBTQIA+: Two-Spirit, Lesbian, Gay, Bisexual, Transgender, Queer, Intersex, Asexual/Aromantic, with the + signifying the ongoing evolution of the understanding of sexuality and gender
